# Methodological bias associated with soluble protein recovery from soil

**DOI:** 10.1038/s41598-018-29559-4

**Published:** 2018-07-25

**Authors:** Lucy M. Greenfield, Paul W. Hill, Eric Paterson, Elizabeth M. Baggs, Davey L. Jones

**Affiliations:** 1 School of Environment, Natural Resources & Geography, Bangor University, Gwynedd, LL57 2UW UK; 2 The James Hutton Institute, Craigiebuckler, Aberdeen, AB15 8QH UK; 3 The Royal (Dick) School of Veterinary Studies, University of Edinburgh, Easter Bush Campus, Midlothian, EH25 9RG UK

## Abstract

Proteins play a crucial role in many soil processes, however, standardised methods to extract soluble protein from soil are lacking. The aim of this study was to compare the ability of different extractants to quantify the recovery of soluble proteins from three soil types (Cambisol, Ferralsol and Histosol) with contrasting clay and organic matter contents. Known amounts of plant-derived ^14^C-labelled soluble proteins were incubated with soil and then extracted with solutions of contrasting pH, concentration and polarity. Protein recovery proved highly solvent and soil dependent (Histosol > Cambisol > Ferralsol) and no single extractant was capable of complete protein recovery. In comparison to deionised water (10–60% of the total protein recovered), maximal recovery was observed with NaOH (0.1 M; 61–80%) and Na-pyrophosphate (0.05 M, pH 7.0; 45–75% recovery). We conclude that the dependence of protein recovery on both extractant and soil type prevents direct comparison of studies using different recovery methods, particularly if no extraction controls are used. We present recommendations for a standard protein extraction protocol.

## Introduction

Protein represents the dominant form of organic nitrogen (N) entering soil ecosystems and frequently the bottleneck in soil N cycling^1^. Further, based on the number of proteins contained in plants and microorganisms, it can be expected that a single gram of soil may contain thousands of different proteins^2,3^. As proteins play a key role in many soil processes, there is increasing interest in the extraction, separation, identification and quantification of proteins as indicators of soil function. However, the development of exoproteomic approaches are currently limited by the lack of standard protocols and the difficulty of recovering proteins from soil.

Extractants that have commonly been used for soil protein recovery include simple salts (e.g. K_2_SO_4_, Na-pyrophosphate, Na-phosphate), bases (e.g. NaOH), organic acids (e.g. Na-citrate) and surfactants (e.g. Tris-SDS) (Supplementary Table S1). Although previous studies have examined a range of protein extraction methods, these have been largely restricted to single unrepresentative proteins (e.g. BSA), single soils or have used quantification methods known to suffer from severe interference by the co-extraction of humic substances^4–6^. In addition, many of these studies have lacked the appropriate controls, preventing determination of protein extraction efficiency or have focused on the whole soil metaproteome.

Soil type has a large influence on protein recovery. Some studies suggest that organic matter and clay content are the key soil properties which affect protein recovery^5,7,8^ whilst other studies suggest soil pH is also important^9–11^. Organic matter content, clay content and pH influence the adsorption of protein in soil and, therefore, affects the ease to which it can be extracted.

Our aim was to focus on soluble proteins and to compare the recovery of a mixture of ^14^C-labelled plant proteins from soil using 39 different extractants. Our secondary aim was to evaluate the influence of soil type on protein recovery.

## Materials and Methods

### Soils used in the study

We evaluated protein recovery from three soils with contrasting organic matter and Fe contents: (1) a Eutric Cambisol obtained from a temperate *Lolium perenne* L. grassland in Abergwyngregyn, Gwynedd, UK (53°14′N, 4°00′W); (2) a Fibric Histosol obtained from a temperate *Calluna vulgaris* (L.) Hull moorland in Abergwyngregyn, Gwynedd, UK (53°22′N, 4°01′W), and (3) a Rhodic Ferralsol obtained from a *Saccharum officinarum* L. plantation in Piracicaba, Brazil (22°32′S, 49°20′W)^12^. In all cases, replicate batches of soil (*n* = 3) were collected from a depth of 0–15 cm, sieved (<2 mm) and kept at 4 °C until required. The main soil properties are shown in Table 1. Soil pH and electrical conductivity (EC) were measured in 1:5 (v/v) soil:H_2_O extracts. Total C and N were determined with a TruSpec^®^ analyser (Leco Corp., St Joseph, MI). Soil texture was determined with a LS1330 Particle size analyser (Beckman Coulter, Brea, CA). Cation exchange capacity (CEC) was measured by saturation with an index cation^13^. Soluble protein in water extracts was measured using the Coomassie Blue method^14^ and was used to calibrate the rate of ^14^C-labelled protein addition (Supplementary Table S3). This method, however, cannot be used with other extractants other than water due to bias from interfering substances^6,15^.

**Table 1:** Major characteristics of the three soils used in the extraction trial.

	Cambisol	Ferralsol	Histosol
pH	6.07 ± 0.02^a^	4.75 ± 0.22^b^	4.48 ± 0.17^b^
EC (µS cm^−1^)	21.8 ± 3.3^a^	140.7 ± 52.9^a^	47.5 ± 28.4^a^
Organic C (%)	2.43 ± 0.02^a^	1.45 ± 0.08^a^	23.2 ± 0.5^b^
Total N (%)	0.21 ± 0.00^a^	0.12 ± 0.00^b^	1.12 ± 0.04^c^
Sand (%)	40.7 ± 3.2^a^	31.0 ± 1.8^a^	90.7 ± 2.4^b^
Silt (%)	46.0 ± 2.7^a^	35.1 ± 2.2^b^	8.0 ± 1.9^c^
Clay (%)	13.3 ± 0.5^a^	33.8 ± 0.7^b^	1.3 ± 0.5^c^
Cation exchange capacity (mmol kg^−1^)	145 ± 6^a^	90 ± 8^a^	334 ± 57^b^

Values represent means ± SEM (Standard error of the mean) (*n* = 3). Different letters (^a,^ ^b,^ ^c^) indicate significant differences between soils at the *p* < 0.05 level.

### Protein extraction solutions

The extractants tested were based on previously published methods (Supplementary Table S1) and included: deionised water, Na-pyrophosphate (0.01, 0.05, 0.1 M; pH 7.0), Na-citrate (0.01, 0.05, 0.1, 0.5 M; pH 8.0), Tris-SDS (0.01, 0.05, 0.1 M SDS with 0.05 M Tris; pH 7.0), K-phosphate buffer (0.01, 0.05, 0.1, 0.5 M; pH 6.0 and 8.0), CaCl_2_, NaOH and K_2_SO_4_ (0.01, 0.05, 0.1, 0.5 M), methanol and ethanol (25%, 50%, 75%, 100% v/v). Extractants with no pH value stated were not adjusted and their values are presented in Supplementary Table S4.

### Protein addition and recovery from soil

Soil (1 g) was placed in individual 20 ml polypropylene vials and heat-sterilised (80 °C, 1 h) immediately prior to experimentation^16^. This sterilisation procedure was not found to affect the CEC of the soils (Supplementary Table S2). In addition, it also proved effective at killing the microbial community preventing bias from microbial breakdown/immobilisation of the added protein (Supplementary Fig. S1). Although free protease activity was not completely eliminated by heat sterilisation, the exoenzyme activity was extremely low compared to the amount of protein added to the soil and was therefore not expected to bias our findings (Supplementary Table S5). Purified, ^14^C-uniformly labelled soluble protein from *Nicotiana tabacum* L. leaves (100 µl; 0.860 mg ml^−1^; 1.2 kBq ml^−1^; purified to >3 kDa by ultra-filtration; custom synthesised by American Radiolabeled Chemicals, St Louis, MO) was added to each soil, shaken to mix and incubated for 30 min at 20 °C. An incubation time of 30 min was deemed appropriate based on initial pilot studies of protein sorption and recovery from soil at incubation times varying from 0.5 to 24 h (Supplementary Table S6). The time is therefore sufficient to obtain high rates of sorption while minimising the chances of proteolysis or microbial regrowth. Soluble plant proteins were chosen as they represent one of the major forms of dissolved organic N added to soil. Based on extractant methods from previous studies, the soils were subsequently shaken with 5 ml of each extractant (30 min; 200 rev min^−1^)^17,18^, then a 1.5 ml aliquot was pipetted into 1.5 ml microfuge tubes and centrifuged (18 000 *g*; 60 s) and the supernatant recovered. The centrifugation time of 60 s allowed complete phase separation of the soil particles and supernatant (Supplementary Table S7). The amount of ^14^C-label recovered in degradations per minute (DPM) of supernatant was determined using a Wallac 1414 scintillation counter (60 s) and Wallac Optiphase HiSafe3 scintillation fluid (PerkinElmer Inc., Waltham, MA). Baseline ^14^C-labelled protein was determined by counting 100 µl of ^14^C-labelled protein. Extraction efficiency was calculated by Equation (1).

1 $$Extraction\,efficiency\,( \% )=\frac{{}^{14}C\,in\,extractant\,supernatant\,(DPM)}{baseline{}^{14}C\,(DPM)}\times 100$$

Humic acids and organic solvents had no effect on ^14^C counting efficiency (Supplementary Tables S8 and S9). To estimate the amount of humic substances co-extracted with the protein, the colour of the extracts was determined at 254 and 400 nm in UV-transparent plastic 96-well plate using a PowerWave HT Spectrophotometer (BioTek Inc., Winooski, VT).

### Statistical analysis

All experiments were performed in triplicate. All statistical analysis was performed using R 3.4.1 and work was carried out in base R unless stated^19^. Data was declared to be normally distributed by Shapiro-Wilk normality test (*p* > 0.05) and have equal variances across groups by Bartlett test (*p* > 0.05). Graphs were created using the R package ggplot2^20^. Differences in soil properties between soil types were analysed by one-way ANOVA with TukeyHSD post-hoc testing using *p* < 0.05 as the cut-off for statistical significance. Differences in protein recovery between treatments and soils were analysed by two-way ANOVA with TukeyHSD post-hoc testing using *p* < 0.05 as the cut-off for statistical significance. Chemical speciation modelling to estimate the net valency of each extractant was performed with Geochem-EZ^21^.

### Data availability

Please contact the corresponding author (l.greenfield@bangor.ac.uk) for access to data.

## Results and Discussion

### Protein recovery from soil by water

**Figure 1:**
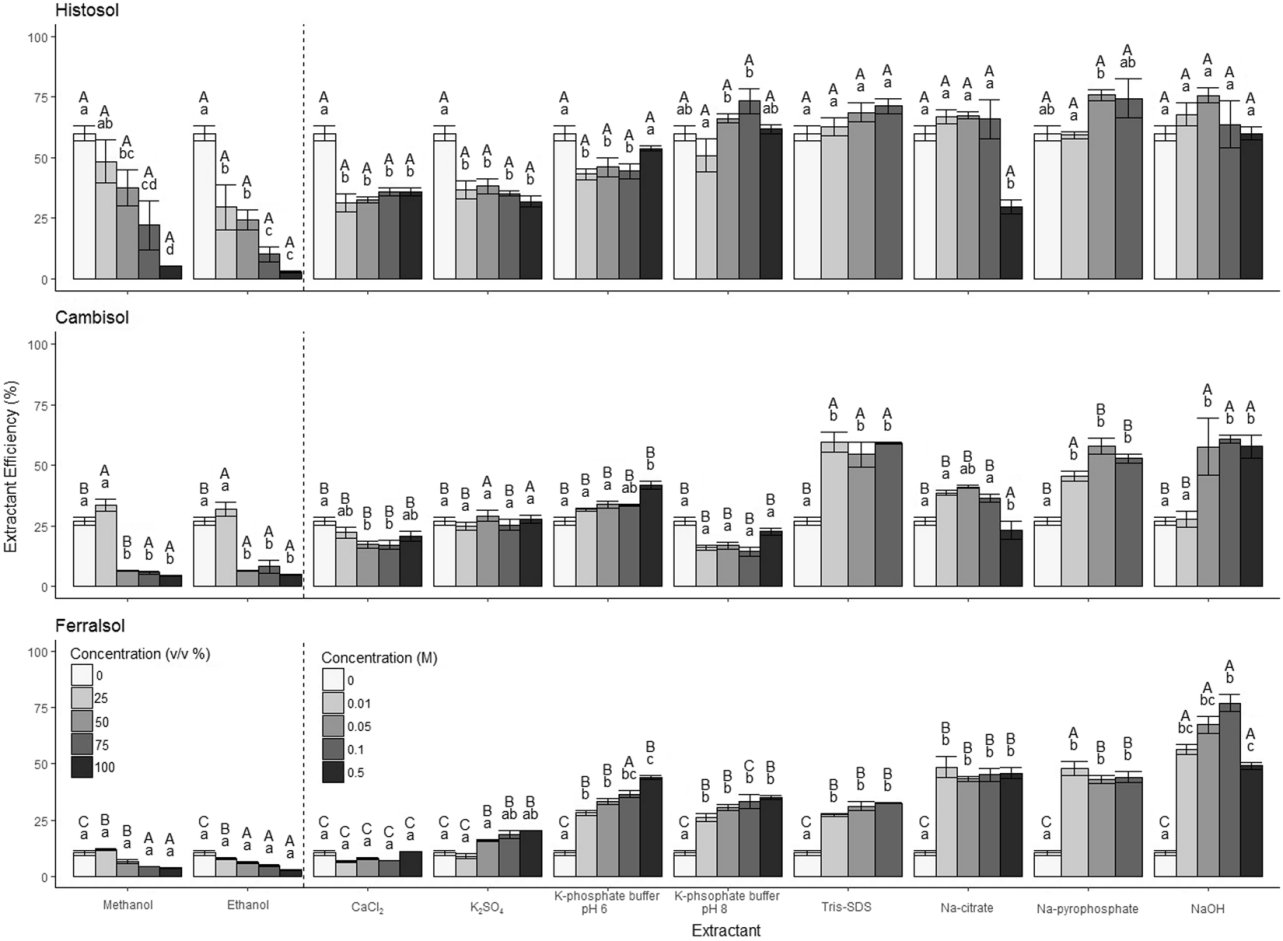
Extraction efficiency (%) of ^14^C-labelled protein from three contrasting soils using a range of chemical extractants. The legend to the left of the dashed line refers concentrations of methanol and ethanol. For all other extractants, refer to the legend on the right of the dashed line. Different capital letters represent significant differences between soil type of the same molarity and extractant. Different letters represent significant differences between molarity of the same soil type and extractant. Values represent means ± SEM (*n* = 3).

Here we aimed to evaluate methods of soluble protein recovery. This is relevant to studies investigating the potential behaviour of isotopically labelled proteins in soil (sorption, biodegradation) or for recovering the plant or microbial exoproteome. Overall, we found significant differences in protein extraction efficiency between the different extractants (F_10_, _318_ = 118.5; *p* < 0.001; Fig. 1) and soils (Histosol > Cambisol > Ferralsol) (F_2_, _318_ = 148.4; *p* < 0.001; Fig. 1). As the soil was sterilised to limit microbial activity^16^, ^14^C measured is assumed to represent intact ^14^C-protein, therefore we refer to extraction efficiency as protein recovered. Protein recovery by deionised water varied from 10–60% between soil types. As the water is expected to recover mainly free, unbound protein, we assume the remainder became bound to the solid phase or coagulated/precipitated on entering the soil^22,23^. Proteins are known to readily sorb to the surface of clay minerals, Fe/Al oxyhydroxides and humic materials in soil^24–26^. Therefore, extractants should be able to displace proteins bound to surfaces during the extraction process or to solubilise the binding surfaces themselves. Ferralsols had the lowest protein recovery probably because of the higher clay and Fe-oxide fraction, compared to the Histosol and Cambisol (*p* < 0.001; Table 1), resulting in more protein being strongly bound to the solid phase. In comparison, the higher humic content of the Histosol (*p* < 0.001; Table 1) may have resulted in the extraction of protein as soluble humic-protein complexes^27^. In our soils, a complete recovery of the added ^14^C-protein was not achieved for any soil, with ca. 25% not recoverable by any extractant. This is likely to be even higher in soils where proteins have been stabilised for long periods.

### Protein recovery from soil by salt extracts

For the Histosol, no significant difference was observed between deionised water and the other extractants (*p* > 0.05) except CaCl_2_ and K_2_SO_4_ which lowered protein extraction compared to deionised water (*p* < 0.05). We ascribe the poor protein recovery with CaCl_2_ and K_2_SO_4_ to salt-induced conformational changes in protein structure and subsequent coagulation/precipitation (Supplementary Table S10), a phenomenon which is well documented in the literature^23^. In contrast to the Histosol, deionised water gave low protein recovery rates from the Ferralsol and Cambisol likely due to more protein adsorbed onto the clay fraction. We conclude therefore that water extracts may provide an estimate of free, unbound proteins in soil and limited information of the bound fraction. Further, while 0.5 M K_2_SO_4_ is frequently used as a standard extractant for dissolved organic N and for measuring soil microbial biomass-N^17,28^, our results suggest that the method may reduce total protein recovery.

The highest recoveries were obtained by NaOH and Na-pyrophosphate (70–76% of the total protein added), with no significant difference apparent between them (*p* > 0.05; Fig. 1). The high pH of NaOH relative to the other extractants solubilises organic matter leading to the release of protein particularly the case of the Histosol^29^. For the Ferrasol, NaOH was the most efficient extractant (49–77% compared to 43–48% by Na-citrate). NaOH also solubilises protein adsorbed to Al(OH)_3_, resulting in protein release from the Ferralsol^30^.

Our results therefore suggest that the recovery of protein from soil is consistent with (i) their salting-out potential based on the Hofmeister series^23^, and (ii) the potential of each salt to displace bound protein from surfaces via ligand exchange, based on their net valency (i.e. HP_2_O_7_^3-^ > Citrate^3-^ > phosphate^1.87-^_(pH 8)_ = phosphate^1.15-^_(pH 6)_ > SO_4_^2-^ > Cl^-^). The exception to this was Tris-SDS^0.09-^ which had a significantly higher extraction efficiency than K_2_SO_4_ and CaCl_2_ (*p* < 0.001) suggesting that the presence of surfactant aids ionic displacement. Surfactants tend to gather around interfaces (e.g. the interface between the soil surface and soil solution). The surfactants compete with the protein molecules for available surface area in order for the hydrophobic tails to avoid water. Over time the SDS molecule will replace the protein molecules because the surfactant molecules are in excess^31,32^.

### Protein recovery from soil by organic solvents

The polar solvents, methanol and ethanol both proved ineffectual at recovering soluble proteins from soil likely due to the alcohol-induced precipitation of proteins^33^. This contrasts strongly with metabolomic studies where these extractants often yield the greatest recovery of low molecular weight organic solutes^34,35^.

### Co-extraction of humic substances

NaOH caused the solubilisation of large amounts of humic substances and based on previous studies, this is likely to induce protein denaturation^36,37^. Consequently, we would not recommend it as an extractant. However, in some analysis the structure of the protein is not important (e.g. sodium dodecyl sulfate-polyacrylamide gel electrophoresis (SDS-PAGE) and the Kjeldahl method) and NaOH can be used.

Na-pyrophosphate, NaOH, Na-citrate and both phosphate buffers extracted more humic substances in comparison to deionised water (Supplementary Table S11; Supplementary Fig. S2) in support of previous findings^11,38^. Humic substances can be problematic due to their ability to bind to proteins and interfere with colorimetric procedures for quantifying protein^4,39^. Proteins interact with humic substances to form protein-humic substance complexes^26^. The mechanisms of the interaction are thought to consist of: (a) covalent and hydrogen bonds^40^, (b) ionic bonds between the functional amino group of the protein and the carboxyl or hydroxyl group of the humic substance^41^, (c) physically immobilised within macromolecular matrix of humic substances^42^, and (d) electron donor-acceptor complexes^43^.

The co-extraction of humic substances with proteins in the protein-humic complexes results in colour in the supernatant. This interferes with colorimetric and fluorescent analysis of protein quantity^6,44^. Methods of removing interfering humic substances (e.g. PVPP^4^ and TCA precipitation^45^) have been found to be ineffective^46^. Therefore, NaOH, Na-pyrophosphate, Na-citrate and phosphate buffers are not ideal extractants when these types of analysis are being used. In addition, if extracting protein from a soil with high organic matter content, more interference will occur in comparison to soils with lower organic matter contents.

## Conclusions

In summary, we found that  0.1 M NaOH was the most effective extractant overall when denatured protein can be used in subsequent analysis and co-extraction of humic substances does not interfere. For analysis of intact proteins, 0.05 M Na-pyrophosphate (pH 7.0) was most effective for extracting water-soluble proteins from soil; however, it did also co-extract humic substances. Where interference of humic substances may prove problematic for subsequent analysis and intact proteins are required, deionised water is recommended. For proteomics, further analysis by LC-MS/MS will be necessary to assess the quality of the proteins extracted by each method^15,47^. In addition, although this study was limited to three soils, our results clearly indicate that soil type directly affects the amount of protein that can be recovered. This may make quantitative comparisons between soils problematic. Rarely has this been accounted for in previous studies comparing protein levels in soil. The impact of this in future studies can be evaluated by measuring the recovery of a known mixture of proteins, as undertaken here. It should also be emphasised that this study focused only on the recovery of hydrophilic proteins from soil. Similar studies are therefore required to optimise the recovery of proteins contained within the soil microbial community, especially those of a hydrophobic nature.

## Electronic supplementary material

Supplementary Information
