# Supplementary material for: Methodological bias associated with soluble protein recovery from soil
Source: Sci Rep. 2018 Jul 25;8:11186. doi: 10.1038/s41598-018-29559-4 (PMC6060134; doi:10.1038/s41598-018-29559-4)
Supplement: Supplementary file 1 — Supplementary Information [file 41598_2018_29559_MOESM1_ESM.docx]

**On-line Supplementary Information**

Methodological bias associated with soluble protein recovery from soil

Lucy M. Greenfield, Paul W. Hill, Eric Paterson, Elizabeth M. Baggs, Davey L. Jones

**S1. Addition supporting material for the manuscript**

*S1.1. Methods for protein recovery from soil*

Table S1 below summarises the wide range of methods used to extract proteins from soil. We hypothesised that the choice of method may greatly affect the amount of protein that could be recovered from soil; however, no one has previously compared the efficiency of these different methods in a systematic way. We chose three contrasting soils to test these methods. We acknowledge that there are many other soil types that could also have been used in our experimentation (e.g. andosols, arenosols, vertisols, calcisols), however, we felt out choice was sufficient to test the general principles associated with protein extraction efficiency.

**Table S1.** Common extractants and methods used for protein recovery from soil. Key results are given for studies that measured extraction efficiency or the effect of soil type on protein recovery.

| **Study** | **Extractants used** | **Method** | **Soil type (classification)** | **Key results** |
| --- | --- | --- | --- | --- |
| ^1^ Friedel & Schelller (2002) | 0.5 M K_2_SO_4_ | - Extracted in 1:2.5 w/v ratio, shaken and centrifuged. - Chloroform-fumigation of extracts - Proteins acid hydrolysed using 10 ml 6 M HCl - Hydrolysed amino acids analysed by fluorescence | Haplic Luvisol  Calcaric Regosol  Fibric Histosol  Haplic Podsol  Mollic Planosol (FAO) |  |
| ^2^ Haney et al. (2001) | 0.001, 0.01, 0.01 and 0.5 M K_2_SO_4_ (pH 6.5) | - Chloroform-fumigation - Extracted with K_2_SO_4_, shaken for 1 h, and then centrifuged - Soil microbial biomass C (SMBC) measured by carbon analyser | Typic Quartzipsamments  Typic Kanhapludults  Torretic Paleustolls  Plinthic Paleudults  Udic Paleustalfs  Oxyaquic Hapluderts  Udifluventic Ustocherpts  Udertic Haplustolls  Typic Calciustolls (USDA) | Decreasing extractant molarity resulted in more SMBC in soils with low pH and lower SMBC in soils with high pH. |
| ^3^ Masciandaro et al. (2008) | 0.1 M Na-pyrophosphate (pH 7)  67 mM phosphate buffer (pH 6)  0.5 M K_2_SO_4_ (pH 6.6) | - Na-pyrophosphate extracted at 1:5 w/v ratio at 37°C and shaken for 24 h. Phosphate buffer and K_2_SO_4_ extracted at 1:3 w/v ratio at room temperature and shaken for 1 h. Both centrifuged. - Proteins analysed by tyrosine and tryptophan determination with Folin–Ciocalteu's reagent and SDS-PAGE | Lithic Calcixeroll  Inceptisol  (FAO) | Overall K_2_SO_4_ extracted more protein.  Extracted protein differed between the two soils.  Similar pattern of proteins found with phosphate buffer and K_2_SO_4_ but pyrophsopahte differed. |
| ^4^ Makarov et al. (2015) | 0.05 and 0.5 M K_2_SO_4_ | - Extracted in 1:5 w/v ratio and shaken for 1 h then centrifuged. - Chloroform-fumigated - Organic C/N and microbial C/N measured | Luvisol  Chernozem  Kastanozem  Umbrisol (FAO) | 0.5 M K_2_SO_4_ extracted more organic C/N but similar quantities of microbial C/N.  The highest amounts of organic C/N and microbial C/N were extracted from Umbrisol. |
| ^5^ Bremner & Lees (1949) | Sodium salts of inorganic acids including 0.1 and 0.5 M pyrophosphate and 0. 5 M chlroide  Sodium salts of organic acids including 0.2 M citrate  0.5 M sodium hydroxide | - Extracted in 1:5 w/v ratio and shaken then centrifuged. - N extracted determined by the micro-Kjeldahl method | Endoleptic Regosol  Chromic Luvisol  (FAO) | More N extracted by 0.5 M Na-pyrophosphate compared 0.1 M Na-pyrophosphate.  Extraction of N differed with soil type and extractant. |
| ^6^ Nannipieri et al. (1974) | 0.1 M Na-pyrophosphate (pH 7.1) | - Extracted in 1:10 w/v ratio, shaken then centrifuged for 30 min at 18,000 *g* - Urease activity measured | Humic podzol  (FAO) |  |
| ^7^ Busto & Perez-Mateos (1995) | 0.01, 0.05, 0.1, 0.2 M Na-pyrophosphate (pH 5-9) | - Extracted in 1:2 or 1:4 w/v ratio, shaken and centrifuged - β-glucosidase activity measured by colorimetric assay | Umbric Dystrochrept  (USDA) | β-glucosidase activity decreased with increasing molarity of Na-pyrophosphate.  Highest β-glucosidase activity was between pH 7 and 8. |
| ^8^ Bonmati et al. (1998) | 140 mM Na-pyrophosphate (pH 7.1) | - Extracted in 1:10 w/v ratio and shaken for 24 h at 37°C then centrifuged for 30 min at 18,000 *g* - Protease activity assayed with Folin–Ciocalteu's reagent - Soil extracts analysed by pyrolysis-gas chromatography | Calcaric Fluvisol  Dystric Cambisol | Soils extracted differing amounts of proteins especially glycoproteins. |
| ^9^ Criquet et al. (2002) | 0.1 M CaCl_2,_ 0.1 M Na-pyrophosphate, 0.1 M Na-citrate, 0.1 M phosphate buffer, 0.1 M *bis*-TRIS (all at pH 6.0) | - Extracted in 1:8.75 w/v ratio and shaken for 6 h at room temperature then centrifuged for 20 min at 12,000 *g* - Protein concentration was measured by the Bradford method | Evergreen oak litter | Highest protein concentrations extracted by *bis*-TRIS and Na-pyrophosphate.  Lowest protein concentrations extracted by CaCl_2_ but had the least amount of interfering substances. |
| ^10^ Halvorson et al. (2006) | 50 mM Na-citrate, 50 mM Na-pyrophosphate, 50 mM Na-oxalate, 50 mM, Na-formate and 50 mM Na-orthophosphate (all at pH 8.0) | - Protein concentration measured by the Bradford method | Ultic Dystrudepts  Typic Paleudults  Typic Hapludalfs  Typic Dystrudepts (USDA) | Highest protein concentrations extracted from soils with more organic matter.  Na-pyrophosphate and Na-citrate extracted the most protein. |
| ^11^ Bastida et al. (2018) | Modified universal buffer (MUB), composed of tris (hydroxymethyl) aminomethane, maleic acid, [citric acid](https://www.sciencedirect.com/topics/earth-and-planetary-sciences/citric-acid), [boric acid](https://www.sciencedirect.com/topics/earth-and-planetary-sciences/boric-acids), NaOH, HCl and distilled water at pH 6.5.  0.1 M Na-pyrophosphate (pH 7.1) | - Extracted in 1:4 w/v ratio and shaken for 1 h then centrifuged for 15 min at 13,000 rpm. Protein pelleted by TCA - Proteins measured by tryptic digestion of protein pellets followed by mass spectrometric analysis | Gypsic xerosol  Calcaric regosol (FAO) | Higher protein concentration when extracted by Na-pyrophosphate. |
| ^12^ Murase et al. (2000) | 67 mM Na-phosphate buffer (pH 6.0) | - Extracted in 1:3 w/v ratio and shaken for 1 h then centrifuged at 12,000 rpm for 30 min - Protein analysed by SDS-PAGE | Entisol (FAO) |  |
| ^13^ Matsumoto et al. (2000) | 1/15 M phosphate buffer (pH 7.0) | - Extracted in 1:4 w/v ratio and shaken for 1 h then filtered - Protein measured by HPLC analyses and SDS-PAGE | Andosol  Cambisol  Fluvisol  Gleysol  Regosol (FAO) | Highest protein concentrations in the Andosol and lowest in the Regosol. |
| ^14^ Kanerva et al. (2007) | 0.05 M phosphate buffer (pH 6 and 8) and 0.1 M Tris-SDS (pH 6.8) | - Extracted in 1:20 w/v, shaken for 3 h, and then centrifuged for 20 min at 10,000 *g* - BSA added to determine protein recovery - Protein concentration measured by the Bradford method - MALDI-TOF-MS analysis of protein | Haplic Podzol  Stagnic Cambisol  Vertic Stagnosol (FAO) | Protein recovery depended on extractant and soil type.  Overall, sandy soils had highest protein recoveries. |
| ^15^ Benndorf et al. (2007) | 0.1 M NaOH | - Extracted in 1:2 w/v ratio, shaken for 30 min, and then centrifuged for 10 min at 16,000 *g* - Humic substances removed by phenol extraction. - Protein analysed by SDS-PAGE | Compost soil |  |
| ^16^ Chen et al. (2009) | 0.05 M citrate (pH 8.0), 0.1 M NaOH, and 0.1 M Tris-SDS (pH 6.8) | - Extracted in 1:10 w/v ratio, shaken for 1 h, and centrifuged for 15 min at 15,000 *g* - Phenol phase to precipitate proteins - Protein analysed by SDS-PAGE | Soils with ranging texture, organic matter and pH | Different protein bands depending on soil type and extractant. |
| ^17^ Keiblinger et al. (2012) | 0.1 M NaOH, 50 mM Tris-SDS and 50 mM Tris-SDS-phenol | - Extracted in 1:3 w/v ratio, shaken for 30 min and centrifuged for 20 min at 3220 *g* - Protein analysed by SDS-PAGE | Dystric Cambisol (FAO)  Standard potting soil | The largest number of proteins was extracted by Tris-SDS-phenol.  More proteins were extracted in Cambisol than potting soil. |
| ^18^ Wright & Upadhyaya (1998) | 20 mM and 50 mM citrate (pH 7.0 and 8.0) | - Extracted in 1:8 w/v ratio, shaken and centrifuged for 5 min at 10,000 *g* - Glomalin measured by enzyme-linked immunosorbent assay | Typic Fragiudults  Typic Dystrochrepts  Mollic Haplaudalfs  Typic Endoaquolls  Aridic Ustochrepts  Calciorthidic Paleustalfs  Typic Upidsamments (USDA) | Amount of glomalin extraction differed with soil type. |
| ^19^ Rosier et al. (2006) | 20 mM Na-citrate (pH 7.0) | - Autoclaved with extractant and then centrifuged at 5000 *g* for 15 min. - Glomalin measured by enzyme-linked immunosorbent assay - BSA added measured by the Bradford method |  | 34-85 % of BSA added recovered. |
| ^20^ Marchetti et al. (2007) | 20 mM Na-citrate (pH 7.0) followed by 50 mM Na-citrate (pH 8.0) | - Extracted in 1:5 w/v ratio, shaken for 6 h, and then centrifuged for 15 min at 10,000 *g* - Cry toxins measured by enzyme-linked immunosorbent assay | Aquic Ustipsamments  Sulfic Endoaquepts (USDA) | Higher extraction of Cry toxins in Sulfic Endoaquepts. |
| ^21^ Schneider et al. (2014) | 50 mM Tris-SDS (pH 7.0) | - Extracted in 1:5 w/v ratio, shaken for 1 h, and then centrifuged for 5 min at 14,000 *g* - Protein analysed by SDS-PAGE | Rendzix Leptosols  Chromic Cambisol  Dystric Cambisol |  |

*S1.2. Cation exchange capacity*

Cation exchange capacity was measured on the fresh soil and heat-sterilised soil (80°C; 1 h). The cation exchange capacity was measured as stated in the main manuscript text ^22^. There were no significant differences between cation exchange capacity in the fresh and heat-sterilised for all of the soil types (Table S2). This suggests that heat sterilisation did not affect cation exchange capacity of the three soils and, therefore, is unlikely to influence the sorption or recovery of proteins by the different extractants.

**Table S2.** Mean cation exchange capacity (mmol kg^-1^ ± SEM) of fresh and heat-sterilised soil for each soil type. Different letters indicate significant differences between treatment for each soil type (paired t test; *p*<0.05).

| Soil Type | Treatment | |
| --- | --- | --- |
|  | **Fresh** | **Heat-sterilised** |
| Cambisol | 145 ± 6^a^ | 104 ± 30^a^ |
| Ferralsol | 90 ± 8^a^ | 72 ± 6^a^ |
| Histosol | 334 ± 6^a^ | 350 ± 6^a^ |

*S1.3. Soluble protein measurements*

We assessed protein content of the soil extractants in 0.5 M K_2_SO_4_ using the Coomassie Blue method^23^. We acknowledge that this method can be subject to some interference e.g. from humic substances ^24^. As Table S3 shows, total protein did not differ significantly between the soil types. Thus ^14^C-labelled protein added to the three soil types was added in a similar ratio. Each gram of soil contained 0.086 mg of ^14^C-labelled protein. Therefore, the rates of ^14^C-labelled protein addition are reflective of natural soil levels.

**Table S3.** Mean soluble protein (mg g^-1^) ± SEM of the three soil types. Different letters indicate significant differences of protein between soil types (one-way ANOVA, Tukey HSD; *p*<0.05).

|  | Soil type | | |
| --- | --- | --- | --- |
|  | **Cambisol** | **Ferralsol** | **Histosol** |
| Soluble protein (mg g^-1^) | 0.035 ± 0.005^a^ | 0.044 ± 0.004^a^ | 0.033 ± 0.013^a^ |

*S1.4. pH of the different extraction solutions*

The extraction efficiency of many inorganic and organic compounds in soil are known to be highly pH dependent. As proteins carry pH dependent charge, it is likely that this may affect their binding to soil particles and, therefore, recovery from soil. Consequently, we measured the pH of our different extraction solutions as shown in Table S4 below.

| **Table S4**. pH of extractants that were not adjusted. | |  |
| --- | --- | --- |
| **Extractant** | **Concentration** | **pH** |
| CaCl_2_ | 0.01 M | 6.07 |
|  | 0.05 M | 6.13 |
|  | 0.1 M | 6.06 |
|  | 0.5 M | 6.39 |
| K_2_SO_4_ | 0.01 M | 5.42 |
|  | 0.05 M | 5.87 |
|  | 0.1 M | 5.49 |
|  | 0.5 M | 5.66 |
| NaOH | 0.01 M | 12.00 |
|  | 0.05 M | 12.70 |
|  | 0.1 M | 13.00 |
|  | 0.5 M | 13.69 |
| Methanol | 25% v/v | 5.45 |
|  | 50% v/v | 5.44 |
|  | 75% v/v | 5.60 |
|  | 100% v/v | 6.88 |
| Ethanol | 25% v/v | 6.59 |
|  | 50% v/v | 6.62 |
|  | 75% v/v | 6.69 |
|  | 100% v/v | 7.51 |

*S1.5. Protein mineralisation*

We have tested the sterility of the soils after heat-sterilisation. Soil (1 g) was sterilised at 80°C for 1 h in 50 ml polypropylene centrifuge tubes. In order to measure the rate of protein breakdown to ^14^CO_2_ ^25^, ^14^C-labelled protein (100 µl; 1.2 kBq ml^-1^) was added to the soil and a NaOH trap (1 M; 1 ml) was placed above the soil and the tubes sealed. At various times after ^14^C-protein addition (0.5, 1, 3, 6 and 24 h) the NaOH trap was taken out and replaced with a fresh trap and the ^14^CO_2_ adsorbed in the NaOH was counted on a Wallac 1404 liquid scintillation counter. Protein mineralisation, as measured by ^14^CO_2_ production, was significantly reduced by heat-sterilisation compared to the fresh soil in all soil types (one-way ANOVA: Cambisol: F_(1,28)_ = 17.7; *p*<0.001, Ferralsol: F_(1,28)_ = 14.6; *p*<0.001, Histosol: F_(1,28)_ = 19.0; *p*<0.001) (Fig. S1). Therefore, heat-sterilisation of the soil was deemed satisfactory for the purposes of our experiment. The results also indicated that minimal microbial activity would have occurred in the 30 min incubation time used in the main protein extraction assays.

**Fig S1.** Cumulative ^14^CO_2_ production after the addition of ^14^C-labelled protein to fresh and heat sterilised soil (means ± SEM, *n* = 3). The legend is the same for all panels.

*S1.6. Protease activity in soil*

Although we have discounted the microbial breakdown of our added ^14^C-labelled protein (see above), it is still possible that our added protein could be broken down into peptide fragments by exoenzymes. To address this, proteolytic activity was measured on the fresh and heat-sterilised soil (80°C; 1 h). Soil was extracted in deionised water (1:5 w/v soil:extractant ratio). An alanine aminopeptidase and leucine aminopeptidase assay was carried out according to Vepsäläinen et al. (2001). Due to the use of deionised water as an extractant, total aminopeptidase activity may be an underestimate. Deionised water may be incapable of removing proteases that have been adsorbed onto soil surfaces (e.g. organic matter and clay minerals). The results demonstrated that protease was very low in the soils relative to the amount of protein we added. However, mild heat sterilisation did not eliminate proteolytic activity (Table S5). Therefore, some very limited proteolysis of ^14^C-labelled proteins may have taken place leading to the production of very small amounts of ^14^C-enriched peptides. We do not expect this, however, to bias our findings. Further, in the main experiment the protein was incubated with soil over 30 min allowing minimal time for proteolysis to take place (especially given the amount of protein added relative to protease activity). This is further supported by the ^14^C mineralisation data presented in Table S5 which shows limited breakdown of the protein even under non-sterile conditions (0.97 ± 0.28% of the total added after 30 min incubation).

**Table S5.** Mean alanine aminopeptidase and leucine aminopeptidase activity (nmol AMC g^-1^ h^-1^) ± SEM (*n* = 3) of fresh and heat-sterilised soil for each soil type. Letters indicate significant differences between treatment for each soil type and assay (paired t test; *p*<0.05).

| Soil type | Alanine aminopeptidase | |  | Leucine aminopeptidase | |
| --- | --- | --- | --- | --- | --- |
|  | **Fresh** | **Heat-sterilised** |  | **Fresh** | **Heat-sterilised** |
| Cambisol | 9.4 ± 0.7^a^ | 8.8 ± 1.1^a^ |  | 8.4 ± 0.5^a^ | 3.8 ± 0.6^b^ |
| Ferralsol | 26.8 ± 11.4^a^ | 9.7 ± 1.9^a^ |  | 7.5 ± 1.1^a^ | 11.9 ± 4.3^a^ |
| Histosol | 9.2 ± 0.8^a^ | 7.8 ± 1.8^a^ |  | 7.5 ± 1.5^a^ | 8.0 ± 1.9^a^ |

*S1.7. Effect of incubation time on protein sorption*

We have tested the effect of a prolonged contact time of the protein with the solid phase and its subsequent recovery. ^14^C-labelled protein (100 µl; 1.2 kBq ml^-1^) was added to 1 g of heat-sterilised soil (80°C; 1 h) and incubated for 30 min, 1 h, 3 h, 6 h and 24 h. After incubation, the ^14^C-labelled protein was extracted by deionised water following the same procedure as described in the main manuscript. ^14^C-labelled protein recovery was unaffected by incubation time for the Ferralsol. Incubation for 24 h reduced recovery relative to the other times for the Histosol and Cambisol (Table S6). We conclude that 0.5 h is an appropriate length of incubation for protein in the soil, limiting protease activity which would cause the ^14^C-labelled protein to be broken down. Longer incubation times (>0.5 h) would have increased the risk of microbial regrowth and exoenzyme degradation of the added protein.

**Table S6.** Effect of incubation time on the recovery of ^14^C-labelled protein from soil (% of total added) mean ± SEM (*n* = 3). Different letters indicate significant differences between extraction efficiencies of different incubation times for each soil type (one-way ANOVA with Tukey HSD; *p*<0.05).

| Soil type | Incubation time (h) | | | | |
| --- | --- | --- | --- | --- | --- |
|  | **0.5** | **1** | **3** | **6** | **24** |
| Cambisol | 26.9 ± 1.8^ab^ | 31.8 ± 2.0^a^ | 31.5 ± 2.2^a^ | 25.0 ± 2.1^ab^ | 18.7 ± 1.4^b^ |
| Ferralsol | 10.4 ± 1.1^a^ | 11.4 ± 0.6^a^ | 11.1 ± 1.3^a^ | 10.1 ± 0.4^a^ | 11.3 ± 0.8^a^ |
| Histosol | 59.9 ± 3.1^a^ | 48.6 ± 7.2^ab^ | 42.4 ± 7.1^ab^ | 39.5 ± 2.1^ab^ | 31.1 ± 3.0^b^ |

*S1.8. Confirmation of effective phase separation by centrifugation*

We used high-speed centrifugation to ensure rapid phase separation of the solid and liquid phases. This protocol followed many previous published studies from our laboratory. However, to confirm this was effective, firstly we extracted the soil with deionised water as described in the main manuscript. We then centrifuged this extract (18 000 *g*; 60 s). To check if any solid remained in the supernatant after centrifugation, we pipetted 0.5 ml of the supernatant into a ceramic crucible. The crucibles were weighed prior to the addition of 0.5 ml of supernatant and then again after 24 h when all the water had evaporated. The maximum weight left in the crucible was 0.0005 g (Table S7) showing quasi-complete phase separation was achieved. Based on the initial weight of soil in the extract we calculate that centrifugation removed 99.80 ± 0.03% of the total solid. This value could be even higher when the weight of dissolved salts in the supernatant are accounted for.

**Table S7.** Crucible weights before and after the addition of 0.5 ml of water extractant supernatant for each soil type.

| Soil type | Repetition | Empty crucible weight (g) | Weight after drying (g) | Difference in weight (g) |
| --- | --- | --- | --- | --- |
| Cambisol | 1 | 24.2103 | 24.2108 | 0.0005 |
|  | 2 | 17.6983 | 17.6985 | 0.0002 |
|  | 3 | 11.5214 | 11.5215 | 0.0001 |
| Ferralsol | 1 | 16.2720 | 16.2721 | 0.0001 |
|  | 2 | 18.9231 | 16.9235 | 0.0004 |
|  | 3 | 18.4251 | 18.4251 | 0.0000 |
| Histosol | 1 | 11.6769 | 11.6770 | 0.0001 |
|  | 2 | 16.1895 | 16.1897 | 0.0002 |
|  | 3 | 12.7913 | 12.7915 | 0.0002 |

*S1.9. Effect of humic acids on ^14^C counting efficiency*

To determine if there was a quenching effect of humic acids in the supernatants of the protein extractants on the counting efficiency of ^14^C within our samples. A stock solution of humic substances (1 g l^-1^) was made using a commercial source of water soluble humic acid (Humic acid-Na salt, Cat. No. H16752; Sigma-Aldrich, Poole, Dorset, UK). From this stock solution, a serial dilution was performed to obtain a range of humic acid concentrations. Two sets of serial dilutions were performed. One dilution series had ^14^C-labelled protein added (100 µl; 114 Bq) to each dilution and the solutions mixed. From this, 0.5 ml was subsequently counted on a Wallac 1404 liquid scintillation counter with automatic quench correction. The quench correction was based on the manufacturer’s algorithm contained within their WinSpectral^®^ software. No significant difference was measured between the activities of samples at different humic acid concentrations showing that humic substances did not affect ^14^C counting efficiency of our added protein (Table S8).

**Table S8**. Mean activity produced by humic acids and humic acids with added ^14^C-labelled protein (Bq) ± SEM (*n* = 3). Different letters indicate significant differences between humic acid concentration for humic acid only dilution and dilution with added ^14^C-labelled protein (one-way ANOVA with Tukey HSD; *p* < 0.05).

|  | Humic acid concentration (g l^-1^) | | | | | |
| --- | --- | --- | --- | --- | --- | --- |
|  | **0** | **0.0625** | **0.125** | **0.25** | **0.5** | **1** |
| Humic acid only | 0.2 ± 0.0^a^ | 0.2 ± 0.3^a^ | 0.2 ± 0.0^a^ | 0.3 ± 0.1^a^ | 0.2 ± 0.1^a^ | 0.2 ± 0.0^a^ |
| + 100 µl ^14^C-labelled protein | 71.8 ± 8.6^a^ | 75.0 ± 9.9^a^ | 77.1 ± 11.6^a^ | 77.4 ± 12.0^a^ | 77.0 ± 10.9^a^ | 74.7 ± 9.8^a^ |

*S1.10. Effect of solvents on ^14^C counting efficiency*

We determined if there was a quenching effect of methanol and ethanol on the counting efficiency of ^14^C within our extracts. In this experiment, we used ^14^C-labelled guanidine as a model compound as it is soluble in water and organic solvents. Mirroring the protocol in the main manuscript, we added a known amount of ^14^C-labelled guanidine (0.36 kBq) to either 0.5 ml of deionised water (control), HPLC-grade methanol or HPLC-grade ethanol. The amount of ^14^C was then subsequently counted on a Wallac 1404 liquid scintillation counter with automatic quench correction. All treatments were run in triplicate. The results showed that there was no significant difference in the measured ^14^C activity for the different solvents showing that they had minimal effect on the ^14^C counting efficiency (one-way ANOVA: F_(2,8)_ = 5.14; *p* = 0.17) as shown below in Table 10 below.

**Table S9.** Mean activity after the addition of ^14^C-labelled guanidine (kBq) ± SEM (*n* = 3) to either water, ethanol or water. Different letters indicate significant differences between the different solvents (one-way ANOVA with Tukey HSD; *p*<0.05).

| **Solvent** | **^14^C content** | **Counting efficiency** |
| --- | --- | --- |
|  | **(kBq sample^-1^)** | **(% relative to water)** |
| **Water** | 0.359 ± 0.007^a^ | 100 ± 1.9 |
| **Ethanol** | 0.369 ± 0.001^a^ | 103 ± 0.4 |
| **Methanol** | 0.370 ± 0.001^a^ | 103 ± 0.3 |

*1.11. Salt induced protein precipitation*

The hypothesis that protein precipitation was induced at high salt concentrations was tested in the absence of soil. ^14^C-labelled tobacco leaf protein (100 µl; 1.2 kBq ml^-1^) was added to deionised water and two simple salt extractants of varying concentration: K_2_SO_4_ (0.01, 0.05, 0.1, 0.5 M) and CaCl_2_ (0.01, 0.05, 0.1, 0.5 M). The solutions were shaken for 30 min (200 rev min^-1^) and then centrifuged for 60 s (18,000 *g*). ^14^C that remained in the solution was determined by liquid scintillation counting as described above. The extraction efficiency (%) is defined as the percentage of ^14^C-labelled protein recovered from the soil after extraction compared to the baseline value of ^14^C-labelled protein added to the soil. Overall, we found significant differences between the extraction efficiency of K_2_SO_4_ and CaCl_2_ in comparison to deionised water (one-way ANOVA: F_(2, 24)_ = 15.63; *p*<0.001) (Table S10). This supports the hypothesis that large salt concentrations cause precipitation of protein is correct.

**Table S10.** The effect of salt concentration on protein recovery from soil. Values represent the mean (*n* = 3) ± SEM.

|  | Concentration (M) | Extraction efficiency (%) |
| --- | --- | --- |
| Deionised water | N/A | 50.4 ± 13.5 |
| K_2_SO_4_ | 0.01 | 21.8 ± 1.1 |
|  | 0.05 | 25.9 ± 1.3 |
|  | 0.1 | 28.1 ± 1.3 |
|  | 0.5 | 28.3 ± 6.3 |
| CaCl_2_ | 0.01 | 17.1 ± 2.6 |
|  | 0.05 | 19.5 ± 1.8 |
|  | 0.1 | 20.1 ± 2.0 |
|  | 0.5 | 24.7 ± 3.0 |

*S1.12. Co-extraction of humic substances during protein recovery*

UV-Visible spectroscopy provides a cheap, quick but reliable proxy of dissolved organic carbon (DOC) concentrations. Absorbance at 254 nm in the UV spectrum detects aromatic humic substances ^27^ and 400 nm detects colour in the visible spectrum ^28^. The ratio of these two wavelengths (E2:E4) provides a measure of humification ^29,30^.

**Table S11**. Mean absorbance (RAU* cm^-1^) ± SEM of supernatant from extractant solutions at 254 nm and 400 nm for 1:100 dilution (n=3)

*Relative absorbance units

|  |  | **Cambisol** | | | **Ferralsol** | | | **Histosol** | | |
| --- | --- | --- | --- | --- | --- | --- | --- | --- | --- | --- |
| Extractant | Concentration (M) | **254** | **400** | **254/400** | **254** | **400** | **254/400** | **254** | **400** | **254/400** |
| Na-pyrophosphate | 0.1 | 0.315 ± 0.009 | 0.041 ± 0.001 | 7.643 ± 0.311 | 0.429 ± 0.059 | 0.048 ± 0.003 | 8.954 ± 1.169 | 0.752 ± 0.127 | 0.146 ± 0.020 | 5.123 ± 0.366 |
|  | 0.05 | 0.204 ± 0.026 | 0.044 ± 0.001 | 4.625 ± 0.493 | 0.466 ± 0.027 | 0.052 ± 0.003 | 9.009 ± 0.166 | 0.578 ± 0.147 | 0.105 ± 0.019 | 5.328 ± 0.560 |
|  | 0.01 | 0.097 ± 0.005 | 0.030 ± 0.0007 | 3.203 ± 0.149 | 0.439 ± 0.044 | 0.067 ± 0.012 | 6.747 ± 0.544 | 0.204 ± 0.044 | 0.044 ± 0.005 | 4.476 ± 0.533 |
| CaCl_2_ | 0.5 | 0.056 ± 0.0003 | 0.027 ± 0.0003 | 2.113 ± 0.039 | 0.169 ± 0.029 | 0.126 ± 0.0003 | 1.341 ± 0.231 | 0.057 ± 0.001 | 0.026 ± 0.0006 | 2.180 ± 0.036 |
|  | 0.1 | 0.058 ± 0.002 | 0.028 ± 0.001 | 2.099 ± 0.030 | 0.180 ± 0.033 | 0.126 ± 0.0006 | 1.426 ± 0.256 | 0.054 ± 0.0003 | 0.026 ± 0.0003 | 2.117 ± 0.024 |
|  | 0.05 | 0.055 ± 0.0006 | 0.026 ± 0.0003 | 2.089 ± 0.013 | 0.299 ± 0.007 | 0.155 ± 0.011 | 1.945 ± 0.098 | 0.055 ± 0.0003 | 0.026 ± 0.0006 | 2.104 ± 0.036 |
|  | 0.01 | 0.056 ± 0.001 | 0.025 ± 0.0003 | 2.212 ± 0.059 | 0.288 ± 0.009 | 0.151 ± 0.020 | 1.954 ± 0.186 | 0.054 ± 0.0003 | 0.026 ± 0.0003 | 2.117 ± 0.024 |
| Na-citrate | 0.5 | 0.310 ± 0.021 | 0.050 ± 0.003 | 6.166 ± 0.239 | 0.381 ± 0.045 | 0.045 ± 0.002 | 8.639 ± 1.406 | 0.854 ± 0.189 | 0.123 ± 0.020 | 6.775 ± 0.640 |
|  | 0.1 | 0.219 ± 0.018 | 0.044 ± 0.002 | 4.924 ± 0.189 | 0.527 ± 0.042 | 0.054 ± 0.003 | 9.757 ± 0.971 | 0.814 ± 0.076 | 0.124 ± 0.011 | 6.561 ± 0.380 |
|  | 0.05 | 0.166 ± 0.049 | 0.037 ± 0.005 | 4.949 ± 1.725 | 0.337 ± 0.060 | 0.055 ± 0.008 | 6.069 ± 0.279 | 0.532 ± 0.152 | 0.093 ± 0.018 | 5.600 ± 0.613 |
|  | 0.01 | 0.076 ± 0.003 | 0.029 ± 0.0007 | 2.650 ± 0.027 | 0.241 ± 0.009 | 0.061 ± 0.006 | 4.114 ± 0.652 | 0.175 ± 0.033 | 0.044 ± 0.004 | 3.882 ± 0.453 |
| K_2_SO_4_ | 0.5 | 0.059 ± 0.000 | 0.027 ± 0.0006 | 2.187 ± 0.047 | 0.058 ± 0.0007 | 0.025 ± 0.0003 | 2.277 ± 0.049 | 0.057 ± 0.0009 | 0.025 ± 0.0003 | 2.238 ± 0.063 |
|  | 0.1 | 0.060 ± 0.002 | 0.028 ± 0.0003 | 2.131 ± 0.083 | 0.064 ± 0.005 | 0.026 ± 0.0006 | 2.481 ± 0.216 | 0.057 ± 0.002 | 0.026 ± 0.0009 | 2.177 ± 0.013 |
|  | 0.05 | 0.059 ± 0.003 | 0.027 ± 0.0003 | 2.172 ± 0.118 | 0.051 ± 0.010 | 0.027 ± 0.0009 | 1.890 ± 0.394 | 0.057 ± 0.0007 | 0.026 ± 0.0003 | 2.152 ± 0.002 |
|  | 0.01 | 0.059 ± 0.002 | 0.028 ± 0.0007 | 2.120 ± 0.023 | 0.053 ± 0.0009 | 0.025 ± 0.0003 | 2.105 ± 0.013 | 0.063 ± 0.001 | 0.027 ± 0.002 | 2.344 ± 0.102 |
| K-phosphate buffer pH 6 | 0.5 | 0.073 ± 0.003 | 0.030 ± 0.002 | 2.410 ± 0.109 | 0.131 ± 0.005 | 0.031 ± 0.0003 | 4.179 ± 0.111 | 0.208 ± 0.049 | 0.044 ± 0.005 | 4.614 ± 0.577 |
|  | 0.1 | 0.063 ± 0.001 | 0.029 ± 0.001 | 2.204 ± 0.088 | 0.110 ± 0.002 | 0.030 ± 0.0003 | 3.615 ± 0.033 | 0.086 ± 0.012 | 0.030 ± 0.002 | 2.828 ± 0.283 |
|  | 0.05 | 0.062 ± 0.001 | 0.027 ± 0.0006 | 2.309 ± 0.014 | 0.079 ± 0.008 | 0.032 ± 0.003 | 2.544 ± 0.448 | 0.082 ± 0.012 | 0.029 ± 0.001 | 2.837 ± 0.298 |
|  | 0.01 | 0.064 ± 0.002 | 0.028 ± 0.0007 | 2.262 ± 0.088 | 0.076 ± 0.009 | 0.028 ± 0.001 | 2.652 ± 0.191 | 0.068 ± 0.005 | 0.029 ± 0.002 | 2.395 ± 0.248 |
| K-phosphate buffer pH 8 | 0.5 | 0.128 ± 0.052 | 0.037 ± 0.008 | 3.218 ± 0.599 | 0.211 ± 0.030 | 0.042 ± 0.005 | 4.996 ± 0.173 | 0.551 ± 0.104 | 0.104 ± 0.013 | 5.184 ± 0.421 |
|  | 0.1 | 0.065 ± 0.002 | 0.028 ± 0.0006 | 2.333 ± 0.050 | 0.133 ± 0.002 | 0.033 ± 0.000 | 4.030 ± 0.063 | 0.356 ± 0.063 | 0.070 ± 0.007 | 4.977 ± 0.451 |
|  | 0.05 | 0.066 ± 0.005 | 0.029 ± 0.002 | 2.261 ± 0.045 | 0.138 ± 0.010 | 0.033 ± 0.001 | 4.213 ± 0.421 | 0.228 ± 0.036 | 0.051 ± 0.002 | 4.404 ± 0.533 |
|  | 0.01 | 0.065 ± 0.004 | 0.028 ± 0.001 | 2.331 ± 0.064 | 0.098 ± 0.009 | 0.035 ± 0.002 | 2.756 ± 0.069 | 0.086 ± 0.008 | 0.029 ± 0.0009 | 2.926 ± 0.230 |
| NaOH | 0.5 | 0.401 ± 0.014 | 0.078 ± 0.003 | 5.116 ± 0.073 | 0.483 ± 0.013 | 0.092 ± 0.004 | 5.260 ± 0.110 | 2.902 ± 0.153 | 0.553 ± 0.035 | 5.262 ± 0.228 |
|  | 0.1 | 0.398 ± 0.017 | 0.088 ± 0.003 | 4.503 ± 0.032 | 0.981 ± 0.152 | 0.240 ± 0.047 | 4.148 ± 0.146 | 1.552 ± 0.309 | 0.346 ± 0.066 | 4.518 ± 0.256 |
|  | 0.05 | 0.274 ± 0.026 | 0.064 ± 0.005 | 4.271 ± 0.122 | 1.252 ± 0.240 | 0.328 ± 0.072 | 3.861 ± 0.101 | 0.960 ± 0.263 | 0.200 ± 0.042 | 4.692 ± 0.322 |
|  | 0.01 | 0.090 ± 0.0009 | 0.031 ± 0.0006 | 2.916 ± 0.052 | 0.284 ± 0.056 | 0.084 ± 0.015 | 3.370 ± 0.078 | 0.191 ± 0.022 | 0.049 ± 0.003 | 3.858 ± 0.229 |
| Tris-SDS | 0.1 | 0.093 ± 0.003 | 0.034 ± 0.0003 | 2.711 ± 0.117 | 0.076 ± 0.002 | 0.030 ± 0.0009 | 2.524 ± 0.130 | 0.126 ± 0.017 | 0.037 ± 0.004 | 3.412 ± 0.182 |
|  | 0.05 | 0.068 ± 0.001 | 0.029 ± 0.0003 | 2.330 ± 0.043 | 0.070 ± 0.003 | 0.030 ± 0.002 | 2.323 ± 0.053 | 0.102 ± 0.009 | 0.035 ± 0.003 | 2.919 ± 0.039 |
|  | 0.01 | 0.085 ± 0.002 | 0.032 ± 0.002 | 2.632 ± 0.125 | 0.058 ± 0.001 | 0.028 ± 0.0003 | 2.108 ± 0.020 | 0.066 ± 0.002 | 0.029 ± 0.001 | 2.319 ± 0.099 |
| Methanol | 100% | 0.054 ± 0.0003 | 0.026 ± 0.000 | 2.064 ± 0.013 | 0.054 ± 0.0003 | 0.026 ± 0.0003 | 2.117 ± 0.024 | 0.080 ± 0.004 | 0.027 ± 0.0003 | 2.984 ± 0.134 |
|  | 75% | 0.056 ± 0.001 | 0.028 ± 0.001 | 1.999 ± 0.120 | 0.056 ± 0.001 | 0.026 ± 0.0006 | 2.141 ± 0.013 | 0.074 ± 0.004 | 0.030 ± 0.002 | 2.468 ± 0.050 |
|  | 50% | 0.056 ± 0.0006 | 0.027 ± 0.0006 | 2.075 ± 0.023 | 0.055 ± 0.001 | 0.027 ± 0.001 | 2.067 ± 0.056 | 0.064 ± 0.002 | 0.026 ± 0.0003 | 2.431 ± 0.079 |
|  | 25% | 0.057 ± 0.002 | 0.028 ± 0.001 | 2.025 ± 0.012 | 0.055 ± 0.0003 | 0.025 ± 0.0003 | 2.158 ± 0.025 | 0.063 ± 0.004 | 0.026 ± 0.0003 | 2.377 ± 0.117 |
| Ethanol | 100% | 0.054 ± 0.0007 | 0.027 ± 0.001 | 1.994 ± 0.066 | 0.054 ± 0.001 | 0.025 ± 0.000 | 2.147 ± 0.048 | 0.083 ± 0.009 | 0.027 ± 0.0006 | 3.051 ± 0.252 |
|  | 75% | 0.055 ± 0.001 | 0.026 ± 0.0009 | 2.091 ± 0.036 | 0.053 ± 0.0003 | 0.025 ± 0.0003 | 2.106 ± 0.036 | 0.076 ± 0.007 | 0.027 ± 0.0007 | 2.842 ± 0.172 |
|  | 50% | 0.061 ± 0.004 | 0.029 ± 0.004 | 2.163 ± 0.159 | 0.054 ± 0.0003 | 0.026 ± 0.0003 | 2.117 ± 0.024 | 0.073 ± 0.003 | 0.027 ± 0.0009 | 2.660 ± 0.096 |
|  | 25% | 0.055 ± 0.0003 | 0.026 ± 0.0006 | 2.104 ± 0.036 | 0.054 ± 0.0009 | 0.025 ± 0.0003 | 2.146 ± 0.049 | 0.066 ± 0.005 | 0.027 ± 0.0003 | 2.475 ± 0.171 |
| Deionised water | 0 | 0.069 ± 0.007 | 0.029 ± 0.002 | 2.341 ± 0.087 | 0.056 ± 0.002 | 0.026 ± 0.001 | 2.131 ± 0.050 | 0.068 ± 0.005 | 0.030 ± 0.002 | 2.296 ± 0.062 |


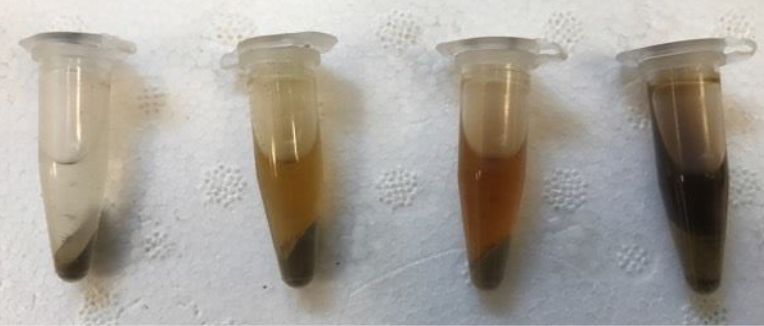


**Fig. S2.** Image to demonstrate colour produced in supernatant from extraction of the Cambisol with different extractants. From left to right: deionised water; 0.5 M Na-citrate; 0.1 M Na-pyrophosphate; 0.5 M NaOH.

**References**

1. Friedel, K., Scheller, E. & Èrgen, J. Composition of hydrolysable amino acids in soil organic matter and soil microbial biomass. *Soil Biol. Biochem.* **34,** 315–325 (2002).

2. Haney, R. L., Franzluebbers, A. J., Hons, F. M., Hossner, L. R. & Zuberer, D. A. Molar concentration of K_2_SO_4_ and soil pH affect estimation of extractable C with chloroform fumigation-extraction. *Soil Biol. Biochem.* **33,** 1501–1507 (2001).

3. Masciandaro, G. *et al.* Comparison of extraction methods for recovery of extracellular β-glucosidase in two different forest soils. *Soil Biol. Biochem.* **40,** 2156–2161 (2008).

4. Makarov, M. I. *et al.* Effect of K_2_SO_4_ concentration on extractability and isotope signature (δ^13^C and δ^15^N) of soil C and N fractions. *Eur. J. Soil Sci.* **66,** 417–426 (2015).

5. Bremner, J. M. & Lees, H. Studies on soil organic matter: Part II. The extraction of organic matter from soil by neutral reagents. *J. Agric. Sci.* **39,** 274–279 (1949).

6. Nannipieri, P., Ceccanti, B., Crevelli, S. & Sequi, P. Use of 0.1 M pyrophosphate to extract urease from a podzol. *Soil Biol. Biochem.* **6,** 359–362 (1974).

7. Busto, M. D. & Perez-Mateos, M. Extraction of humic-β-glucosidase fractions from soil. *Biol. Fertil. Soils* **20,** 77–82 (1995).

8. Bonmati, M., Ceccanti, B. & Nannipieri, P. Protease extraction from soil by sodium pyrophosphate and chemical characterization of the extracts. *Soil Biol. Biochem.* **30,** 2213–2125 (1998).

9. Criquet, S., Farnet, A. M. & Ferre, E. Protein measurement in forest litter. *Biol. Fertil. Soils* **35,** 307–313 (2002).

10. Halvorson, J. J. & Gonzalez, J. M. Bradford reactive soil protein in Appalachian soils: distribution and response to incubation, extraction reagent and tannins. *Plant Soil* **286,** 339–356 (2006).

11. Bastida, F., Jehmlich, N., Torres, I. F. & García, C. The extracellular metaproteome of soils under semiarid climate: A methodological comparison of extraction buffers. *Sci. Total Environ.* **619–620,** 707–711 (2018).

12. Murase, A., Yoneda, M., Ueno, R. & Yonebayashi, K. Isolation of extracellular protein from greenhouse soil. *Soil Biol. Biochem.* **35,** 733–736 (2003).

13. Matsumoto, S., Ae, N. & Yamagata, M. Extraction of mineralizable organic nitrogen from soils by a neutral phosphate buffer solution. *Soil Biol. Biochem.* **32,** 1293–1299 (2000).

14. Kanerva, S., Smolander, A., Kitunen, V., Ketola, R. A. & Kotiaho, T. Comparison of extractants and applicability of MALDI-TOF-MS in the analysis of soil proteinaceous material from different types of soil. *Org. Geochem.* **56,** 1–9 (2013).

15. Benndorf, D., Balckem, G. U., Harms, H. & von Bergen, M. Functional metaproteome analysis of protein extracts from contaminated soil and groundwater. *Int. Soc. Microb. Ecol.* **1,** 224–234 (2007).

16. Chen, S., Rillig, M. C. & Wang, W. Improving soil protein extraction for metaproteome analysis and glomalin-related soil protein detection. *Proteomics* **9,** 4970–4973 (2009).

17. Keiblinger, K. M. *et al.* Soil metaproteomics - Comparative evaluation of protein extraction protocols. *Soil Biol. Biochem.* **54,** 14–24 (2012).

18. Wright, S. F. & Upadhyaya, A. A survey of soils for aggregate stability and glomalin, a glycoprotein produced by hyphae of arbuscular mycorrhizal fungi. *Plant Soil* **198,** 97–107 (1998).

19. Rosier, C. L., Hoye, A. T. & Rillig, M. C. Glomalin-related soil protein: Assessment of current detection and quantification tools. *Soil Biol. Biochem.* **38,** 2205–2211 (2006).

20. Marchetti, E., Accinelli, C. & Epifani, R. Persistence of Cry toxins and cry genes from genetically modified plants in two agricultural soils. *Agron. Sustain. Dev.* **27,** 231–236 (2007).

21. Schneider, T. *et al.* Who is who in litter decomposition? Metaproteomics reveals major microbial players and their biogeochemical functions. *ISME J.* **6,** 1749–1762 (2012).

22. Rhoades, J. D. in *Methods of soil analysis. Part 2. Chemical and Microbiological Properties* (eds. Page, A. L., Miller, R. H. & Keeney, D. R.) 149–157 (Soil Science Society of America, 1982).

23. Bradford, M. M. A Rapid and Sensitive Method for the Quantitation of Microgram Quantities of Protein Utilizing the Principle of Protein-Dye Binding. *Anal. Biochem.* **72,** 248–254 (1976).

24. Roberts, P. & Jones, D. L. Critical evaluation of methods for determining total protein in soil solution. *Soil Biol. Biochem.* **40,** 1485–1495 (2008).

25. Jan, M. T., Roberts, P., Tonheim, S. K. & Jones, D. L. Protein breakdown represents a major bottleneck in nitrogen cycling in grassland soils. *Soil Biol. Biochem.* **41,** 2272–2282 (2009).

26. Vepsäläinen, M., Kukkonen, S., Vestberg, M., Sirviö, H. & Maarit Niemi, R. Application of soil enzyme activity test kit in a field experiment. *Soil Biol. Biochem.* **33,** 1665–1672 (2001).

27. Edzwald, J. K., Becker, W. C. & Wattier, K. L. Surrogate Parameters for Monitoring Organic Matter and THM Precursors. *Res. Technol.* **77,** 122–132 (1985).

28. Wallage, Z. E. & Holden, J. Spatial and temporal variability in the relationship between water colour and dissolved organic carbon in blanket peat pore waters. *Sci. Total Environ.* **408,** 6235–6242 (2010).

29. Peacock, M. *et al.* UV-visible absorbance spectroscopy as a proxy for peatland dissolved organic carbon (DOC) quantity and quality: considerations on wavelength and absorbance degradation. *Environ. Sci. Process. Impacts* (2014).

30. Carter, H. T. *et al.* Freshwater DOM quantity and quality from a two-component model of UV absorbance. *Water Res.* **46,** 4532–4542 (2012).
